# Pacemaker Dependency after Cardiac Surgery: A Systematic Review of Current Evidence

**DOI:** 10.1371/journal.pone.0140340

**Published:** 2015-10-15

**Authors:** Curtis M. Steyers, Rohan Khera, Prashant Bhave

**Affiliations:** 1 Department of Internal Medicine, University of Iowa Carver College of Medicine, Iowa City, Iowa, United States of America; 2 Division of Cardiovascular Medicine, Department of Internal Medicine, University of Iowa Carver College of Medicine, Iowa City, Iowa, United States of America; Istituto Clinico S. Ambrogio, ITALY

## Abstract

**Background:**

Severe postoperative conduction disturbances requiring permanent pacemaker implantation frequently occur following cardiac surgery. Little is known about the long-term pacing requirements and risk factors for pacemaker dependency in this population.

**Methods:**

We performed a systematic review of the literature addressing rates and predictors of pacemaker dependency in patients requiring permanent pacemaker implantation after cardiac surgery. Using a comprehensive search of the Medline, Web of Science and EMBASE databases, studies were selected for review based on predetermined inclusion and exclusion criteria.

**Results:**

A total of 8 studies addressing the endpoint of pacemaker-dependency were identified, while 3 studies were found that addressed the recovery of atrioventricular (AV) conduction endpoint. There were 10 unique studies with a total of 780 patients. Mean follow-up ranged from 6–72 months. Pacemaker dependency rates ranged from 32%-91% and recovery of AV conduction ranged from 16%-42%. There was significant heterogeneity with respect to the definition of pacemaker dependency. Several patient and procedure-specific variables were found to be independently associated with pacemaker dependency, but these were not consistent between studies.

**Conclusions:**

Pacemaker dependency following cardiac surgery occurs with variable frequency. While individual studies have identified various perioperative risk factors for pacemaker dependency and non-resolution of AV conduction disease, results have been inconsistent. Well-conducted studies using a uniform definition of pacemaker dependency might identify patients who will benefit most from early permanent pacemaker implantation after cardiac surgery.

## Introduction

Post-operative conduction disorders are a major source of morbidity and mortality after cardiac surgery. The incidence of severe postoperative bradyarrhythmias after cardiac surgery requiring permanent pacing varies with type of surgery and ranges between 0.8% to 24%[1]. Although the prevalence and predictors for postoperative permanent pacemaker (PPM) implantation following cardiac surgery have been well described[2], little is known about long-term pacing requirements in this population.

A proportion of patients receiving permanent pacemakers for postoperative bradyarrhythmias have been observed to spontaneously recover native conduction, obviating the continued need for pacing[3, 4]. Despite this observation, the optimal timing for PPM implantation in the postoperative setting has not been clearly defined, due in part to a poor understanding of the natural history of postoperative conduction disturbances. Optimizing timing and patient selection for insertion of PPM in the postoperative setting might lead to fewer inappropriate device implantations in patients without the need for long-term pacing, thereby reducing the risk of adverse events and substantial financial burden associated with a PPM. There is substantial variability in the existing literature with respect to long-term pacemaker dependency and its diagnostic criteria, incidence, pathophysiological basis and predictive risk factors.

We systematically review the current literature for post-cardiac surgery conduction disease and pacemaker dependency and summarize the available evidence highlighting avenues for further study.

## Methods

We performed a systematic literature review for pacemaker dependency and recovery of native conduction after cardiac surgery. We designed our search strategy and criteria for selection of studies to fulfill the following major objectives. First, we sought to examine how pacemaker dependency was defined in the different studies. Next, in patients requiring PPM implantation after cardiac surgery we aimed to study the prevalence of pacemaker dependency or failure to recover AV conduction at a follow-up of at least 1 month. Finally, we endeavored to examine the preoperative, intraoperative or postoperative factors that independently predicted late pacemaker dependency or failure to recover native conduction in the post-cardiac surgery population. The systematic review was conducted in accordance with the PRISMA guidelines and a checklist for their application in provided in S1 PRISMA Checklist [5].

In order to be all-inclusive, a search for English language articles was performed in Medline, Web of Science and EMBASE and a list of search terms for each database are provided separately (S1 Fig). The studies represented in Table 1 were included if they met the following inclusion criteria: specific assessment for permanent pacemaker (PPM) dependency, age ≥18 years, patients undergoing cardiac surgery via midline sternotomy or minimally invasive surgical techniques, explicit and consistent definition for pacemaker dependency along with information regarding its incidence in the study population. Studies of patients with non-cardiac surgery or percutaneous cardiac procedures were excluded. For Table 2, a similar selection strategy was used and studies were selected if they presented data for recovery of conduction instead of pacemaker dependence. Two independent investigators (CMS and RK) individually reviewed each study for inclusion and exclusion criteria and resolved disagreements by consensus.

**Table 1:** Patient and procedural characteristics in the studies addressing pacemaker dependency after cardiac surgery.

Study (Author, year)	Feldman,1992	Glikson, 1997	Onalan, 2008	Huynh, 2009	Merin, 2009	Raza, 2011	Baraki, 2013	Rene, 2013
**Study characteristics**								
Design, Retrospective (R) or Prospective (P)	R	R	R	R	R	R	P	R
Sample size	36	120	102	15	72	141	138	104
Follow up completed (N)	36	86	102	10	58	90	129	98
Follow-up duration (mean months)	36	41	32	32	72	67	64	43
Mortality	-	38% (46)	-	20% (3)	32% (23)	21% (19)	33% (45)	-
**Patient characteristics**								
Age (mean years)	-	72^a^	68	70	67	69	71	67
Sex (% male)	92% (33)	48% (57)	62% (63)	60% (9)	61% (44)	99% (89)		66% (65)
Prior cardiac surgery	-	19% (23)	8% (8)	-	11% (8)	9% (8)	20% (27)	-
Co-morbid conditions								
CAD	53% (19)	-	65% (66)	20% (3)	49% (21)	39% (55)		-
LVEF <50%	-	-	12% (12)	-	24% (17)	-	38% (52)	-
DM	88% (23)	-	23% (23)	33% (5)	29% (21)	28% (25)	23% (31)	-
Preoperative conduction								
LBBB	-	17% (20)	9% (8)	6.7% (1)	25% (18)	-	9% (13)	11% (11)
RBBB	-	12% (14)	9% (8)	6.7% (1)	15% (11)	-	12% (16)	7% (7)
1st degree AV block	-	36% (46)	13% (12)	13% (2)	14% (10)	34% (31)	14% (19)	-
LAFB	-	15% (18)	7% (6)	0	4% (3)	-	10% (14)	9% (9)
2+ degree AV block	-	6% (7)	-	6.7% (1)	1% (1)	-	7% (10)	0
Normal sinus rhythm	-	13% (16)	-	93% (14)	-	18% (25)	47% (64)	82% (80)
Atrial Fibrillation	-	11% (13)	25% (25)	6.7% (1)	-	14% (20)	17% (23)	-
Medications								
Beta-blocker	0	-	-	47% (7)	51% (37)	-	25% (34)	-
Anti-arrhythmic	-	-	-	-	21% (15)	-	8% (11)	-
**Procedure characteristics**								
Procedure type								
AVR	0	23% (28)	60% (61)	40% (6)	53% (38)	-	100% (138)	72% (71)
MVR	0	5% (6)	23% (23)	0	25% (18)	-	0	11% (11)
Combined AVR/MVR	0	5% (6)	-	13% (2)	-	6% (8)	0	10% (10)
CABG	86% (31)	25% (30)	63% (64)	0	58% (42)	30% (43)	0	33% (32)
Combined valve + CABG	14% (5)	13% (16)	-	47% (7)	-	62% (88)	0	-
Cross-clamp time—minutes (mean)	-	72	-	125	92	121	67	82
On-pump time—minutes (mean)	-	112	-	157	141	168	104	-
**Pacemaker placement**								
Indications								
Complete heart block	72% (26)	54% (65)	70% (71)	87% (13)	82% (59)	55% (78)	75% (103)	100% (98)
Slow atrial fibrillation	0	7% (8)	11% (11)	0	13% (9)	25% (35)	16% (22)	0
Sinus node dysfunction	28% (10)	11% (13)	20% (20)	13% (2)		20% (28)	2% (3)	0
Mobitz II 2^nd^ degree block	0	3% (4)	-	0	3% (2)			0
Timing of PM implantation (median days post-op)	-	11	10	6	13	7	7	6

Reported values represent percentage of patients in each category, with numbers in parentheses, unless specified. Abbreviations: AVR–aortic valve replacement, MVR–mitral valve replacement, CABG–coronary artery bypass grafting, CAD–coronary artery disease, LVEF–left ventricular ejection fraction, DM–diabetes Mellitus, LBBB—left bundle branch block, RBBB–right bundle branch block, PM–pacemaker, preop–preoperative, postop–postoperative

^a^ Median

**Table 2:** Studies addressing recovery of conduction after cardiac surgery.

Study (Author, year)	Zakhia, 1992	Kim, 2001	Simms, 2013	Rene, 2013
**Study characteristics**				
Sample size	29	9	14	104
N completed	25	7	13	98
Follow-up duration (mean months)	35	12	6	43
Mortality n	17% (5)	-	7% (1)	-
**Patient characteristics**				
Age (mean)	65	54	68	67
Sex (%male)	55% (16)	33% (3)	57% (8)	66% (65)
Comorbid conditions				
CAD	-	-	36% (5)	-
LVEF <50%	-	22% (2)	50% (7)	-
DM	-	11% (1)	29% (4)	-
Preoperative conduction				
LBBB	31% (9)	-	-	11% (11)
RBBB	7% (2)	-	-	7% (7)
1st degree AV block	7% (2)	-	-	-
LAFB	3.5% (1)	-	-	9% (9)
2+ degree AV block	0	-	-	0
Normal sinus rhythm	-	-	-	82% (80)
Atrial Fibrillation	-	-	0	-
**Procedure type**				
AVR	79% (23)	44% (4)	100% (13)	72% (71)
MVR	10% (3)	22% (2)	0	11% (11)
Combined AVR/MVR	10% (3)	0	0	10% (10)
CABG	0	0	0	33% (32)
Combined valve+ CABG	7% (2)	33% (3)	0	-
**Pacemaker placement**				
Indications				
Complete heart block n	90% (26)	100% (9)	100% (13)	100% (98)
Slow atrial fibrillation	0	0	0	0
Sinus node dysfunction	0	0	0	0
Mobitz II 2^nd^ degree block	10% (3)	0	0	0
Timing of PM implantation (median days post-operatively)	10	8	6.6	6
**Late recovery of conduction**	**16% (4)**	**29% (2)**	**38% (5)**	**42% (41)**

Reported values represent percentage of patients in each category, with numbers in parentheses, unless specified. All studies were retrospective. Abbreviations: AVR–aortic valve replacement, MVR–mitral valve replacement, CABG–coronary artery bypass grafting CAD–coronary artery disease, LVEF–left ventricular ejection fraction, DM–diabetes Mellitus, LBBB—left bundle branch block, RBBB–right bundle branch block, PM–pacemaker

**Fig 1:**
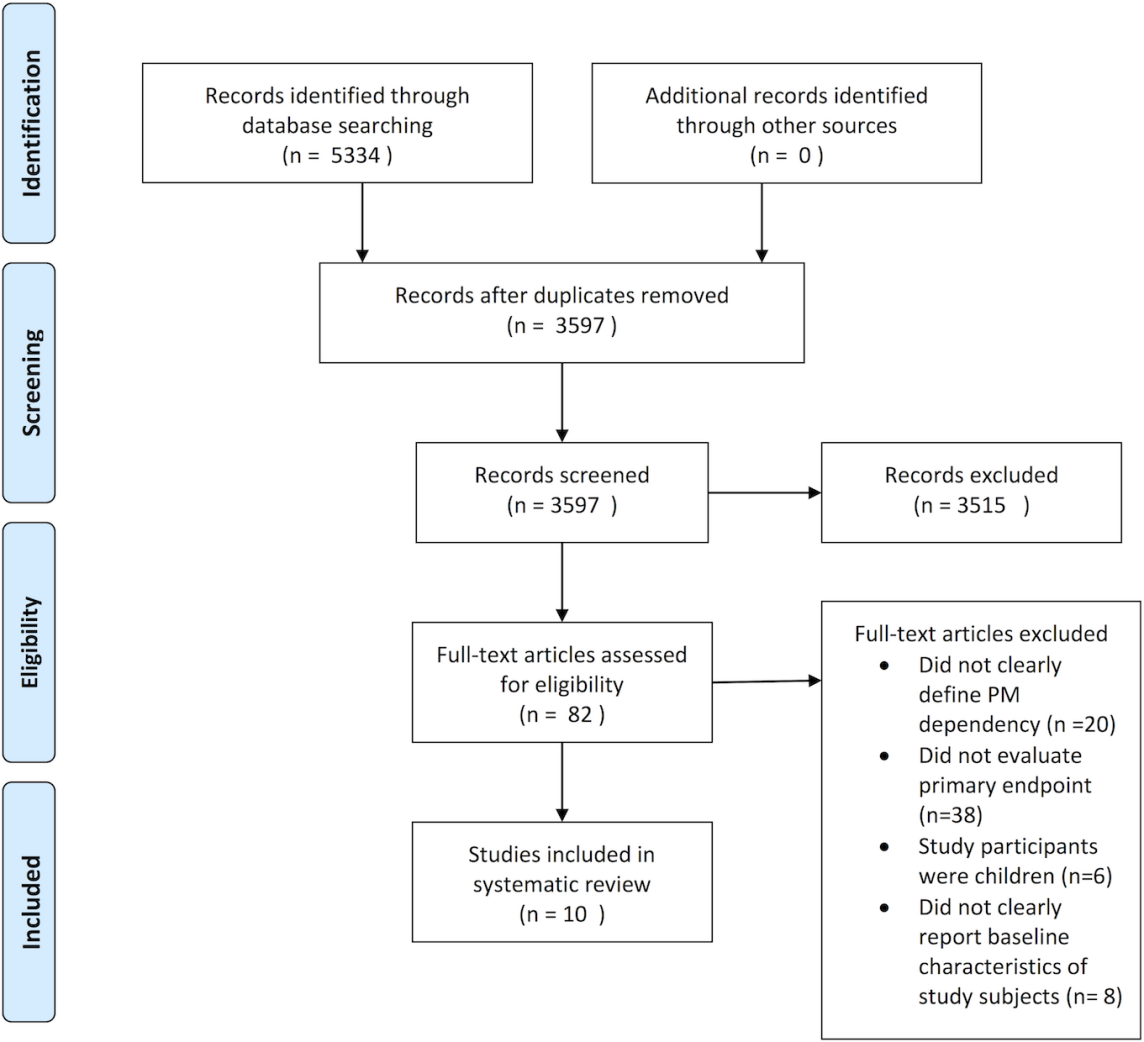
Flowsheet for selection of included studies (PRISMA).

A total of ten studies met the pre-specified inclusion and exclusion criteria and were selected for inclusion in the systematic review (Fig 1). Eight studies specifically addressed the ‘pacemaker dependency’ endpoint and were included in Table 1. Three studies addressed the ‘recovery of native conduction’ endpoint and were included in Table 2. One study documented both endpoints and was included in both tables. All studies are presented chronologically. The references were managed using the Endnote X7 for Mac (Thomson Reuters). Study quality for each of the included studies was assessed using the ‘Good ReseArch for Comparative Effectiveness (GRACE) checklist and is provided as S1 Table [6].

## Results

### Definition

In the selected studies (Table 1), there is substantial variability in the definition for pacemaker dependency (Table 3). Six of the eight included studies used the ‘turn down’ approach of lowering the pacing rate to assess for the underlying rhythm to establish pacemaker dependency. The rate of pacing and duration of observation for the underlying rhythm varied significantly between these studies. Three of these studies used a pacing rate of 30 bpm, whereas another two studies selected a turn-down rate of 40 bpm. Duration of assessment for intrinsic rhythm varied between 10 and 30 seconds. The origin of the detected intrinsic rhythm (i.e. an atrial rhythm with antegrade conduction, junctional escape, or ventricular escape) and the sensitivity for its detection with the use of different pacing rates has not been examined in the selected studies. Hence, the impact of a lower (30 bpm) versus a higher (50 bpm) interrogation rate on the results of the studies is not known. Similarly, the best duration of rhythm assessment has not been validated scientifically. One of the studies used an interrogation-based approach[7], wherein the previous 6 months of pacing data were reviewed by the cardiologist. Dependency was defined as continuous ventricular stimulation without any pacemaker inhibition by spontaneous cardiac activity. Non-dependent patients did not show any pacemaker activity. The authors also assigned patients into an ‘intermittent dependency’ category. These patients showed intermittent ventricular pacing which was quantified as a percentage of all pacemaker stimulation during the preceding 6 month interval. In another study, the authors used a combination of the device turn-down and interrogation approaches to define pacemaker dependency during outpatient follow up[8].

**Table 3:** Definitions of pacemaker (PM) dependency in selected studies.

Study (First author, year)	Definition of PM dependency
Feldman, 1992	Turn-down to 50 bpm with continued paced rhythm, or no intrinsic rhythm during generator change
Glikson, 1997	Paced rhythm with PM set below 50 bpm OR AV delay ≥220 ms in intrinsic rhythm
Onalan, 2008	Turn down to 30 bpm in VVI mode–no intrinsic activity
Huynh, 2009	Turn down to 30 bpm for 30s – absence of escape rhythm >30 bpm
Merin, 2009	Turn down to 40 bpm for 40s – continued paced rhythm at 40 bpm
Raza, 2011	Turn down to 40 bpm for 30s – 100% paced
Baraki, 2013	6 month PM Interrogation—absence of spontaneous cardiac conduction
Rene, 2013	Turn down to 30 bpm for 10s in VVI mode–continued pacing at 30 bpm

Abbreviations: PM—Pacemaker, bpm–beats per minute, VVI—Ventricle paced, ventricle sensed; pacing inhibited if beat sensed, s- seconds, ms—milliseconds

The dependency rates reported in the current literature varied from 32% to 91% with the highest dependency rate obtained using the interrogation approach. Restricting to studies with the turn-down approach was still associated with significant variability in the dependency rates, notably between 32% and 70% (Table 4).

**Table 4:** Pacemaker dependency rates and reported associations in selected studies.

Study (Author, year)	Pacemaker dependent, percent (n)	Significant associations with pacemaker dependency
Feldman, 1992	56% (n = 20)	None
Glikson, 1997	59% (n = 51)^b^	Postoperative complete AV block
Onalan, 2008	32% (n = 33)	1) History of syncope; 2) BMI ≥ 28.5; 3) Bypass time > 105 min; 4) AV block as pacemaker indication
Huynh, 2009	70% (n = 7)	None
Merin, 2009	63% (n = 37)	1) Preoperative LBBB; 2) Persistent postoperative 3rd degree AV block
Raza, 2011	40% (n = 36)	PR interval ≥200 ms on baseline EKG
Baraki, 2013	91% (n = 76)	None
Rene, 2013	45% (n = 44)	Persistent postoperative AV block

^b^ Excludes indeterminate from analysis

Abbreviations: AV–Atrioventricular, LBBB–Left bundle branch block

### Risk factors

Of the 10 included studies, only 4 compared outcomes between patients with and without pacemaker dependency (Table 4). A number of preoperative, intra-operative and post-operative differences were identified by these studies. Feldman et al demonstrated that 17 of the 26 patients who underwent pacemaker placement for postoperative complete heart block were pacemaker dependent at a mean 3 years of follow up[9]. This was defined as the presence of pacemaker activity upon turning down the pacemaker rate to slower than 50 bpm. The patients who were pacemaker dependent were noted to be similar to patients without pacemaker dependency for the reported characteristics, including age (66 years vs. 67 years), sex (94% vs. 100% males), history of prior myocardial infarction (53% vs 55%) and preoperative left ventricular (LV) function (LV ejection fraction, LVEF – 45% vs. 41%). The dependent group had a lower observed rate of preoperative conduction defects (41% vs. 67%), however, these differences were not statistically significant.

Rene et al[4] found that among 98 patients who required pacemaker placement post operatively for 3^rd^ degree AV block after valve surgery only 58% had persistent high-grade AV block after a mean 3.6 years of follow up. Only 45% of patients were pacemaker dependent on follow up, which was defined as presence of paced rhythm with pacemaker programmed to a rate of 30 bpm. Patients with and without late AV block were noted to have similar preoperative characteristics, including age at presentation (67 years in both groups), sex (61% vs. 73%), left ventricular function (LVEF – 52% vs 50%) and baseline conduction (with similar mean PR, RR and QRS intervals; proportion with sinus rhythm – 82% vs. 83% and pre-existing bundle block–LBBB, 11% vs. 12%). Patients with late AV block had similar operative characteristics. Both groups had similar rates of aortic valve surgery (84% vs. 85%), mitral valve surgery (23% vs. 29%) and coronary artery bypass grafting (26% vs. 41%) and had similar average cross-clamp times (82 minutes and 83 minutes). Patients with persistent post-operative AV block, defined as high-grade AV block at each temporary pacemaker evaluation during the immediate in-hospital postoperative period, were more likely to have late pacemaker dependency (55%) when compared to patients without persistent post-operative AV block (25%). Similar comparisons were not reported in other studies.

Raza et al[10] defined pacemaker dependency as presence of 100% paced rhythm upon turning pacemaker down to 40 bpm. Although patients requiring postoperative pacemaker placement after cardiac surgery were older (69 years vs. 67 years), there were no significant differences in patient age, sex (99% male in both), comorbidities (diabetes, hypertension and chronic obstructive pulmonary disease), history of prior myocardial infarction (31% vs. 33%) or heart surgery (11% vs. 7%) in patients with late pacemaker dependency compared to nondependent patients. Patients with dependency also had similar LV function (LVEF 53% vs. 52%) and similar prevalence of class III/IV heart failure (42% vs. 53%). However, in contrast to Rene et al[4], their patient cohort revealed an association between preoperative prolonged PR (> 200 ms) and QRS (>120 ms) intervals and pacemaker dependency, with a higher proportion of pacemaeker dependent patients having prolonged PR and widened QRS intervals (50% for each) compared to nondependent patients (24% and 28%, respectively). They found that the duration of cardiopulmonary bypass predicted both the requirement for post-operative PPM placement and late pacemaker dependency. Pacemaker dependent patients had longer bypass times compared to non-dependent patients (186 minutes vs 152 minutes). While they report an association between longer aortic cross-clamp times and immediate postoperative PPM requirement, however, there was no association with late pacemaker dependency.

Merin et al, who defined pacemaker dependency as continued pacing after turning down the pacemaker to 40 bpm for over 10 seconds, identified preoperative LBBB and Persistent postoperative 3rd degree AV block as independent predictors of PM dependency. While they report that 37 of 58 patients completing follow up were pacemaker dependent, they do not provide a descriptive comparison between the two groups.

## Discussion

A systematic review of the literature regarding conduction disturbances and pacemaker dependency after cardiac surgery reveals several important findings. First, there is substantial variability between existing studies with respect to definitions of pacemaker dependency, and this variability fundamentally limits our ability to draw comparisons between studies. Second, the reported rates for late pacemaker dependency and recovery of native conduction varied widely. Third, although several perioperative patient and procedure-specific factors were identified in individual studies as independent predictors of long-term pacemaker dependency, there was no agreement between studies.

Recovery of native conduction and restoration of a satisfactory and stable intrinsic rhythm after pacemaker implantation can be assessed in several distinct ways. The studies identified herein have framed this endpoint in two general ways: (1) presence or absence of “pacemaker dependency” and (2) recovery of AV conduction. While fundamentally distinct, these two endpoints address one clinically significant question: does the patient continue to require artificial pacing to sustain a hemodynamically adequate rhythm? There is no currently accepted definition of pacemaker dependency. This ambiguity is evident in the studies selected for this review, which have used multiple different sets of criteria for pacemaker dependency. The traditional definition of pacemaker dependency is the absence of an underlying escape rhythm (asystole) after cessation of ventricular pacing. In practice, the absence of an escape rhythm with the pacemaker set at 30–50 beats-per-minute (bpm) in the VVI mode (Ventricle paced, Ventricle sensed, and pacemaker Inhibited in response to a sensed beat) or the presence of symptoms despite an escape rhythm greater than 30–50 bpm in VVI mode is often used to define dependency. The precise rate of ventricular pacing and duration of observation required vary between studies (Table 3). Others have defined pacemaker dependency by quantifying a minimum percentage of paced ventricular events over a preceding interval during device interrogation, using a range of cut-off values to define dependency.

Another major factor in the selected studies is the variability in the chosen period of follow up. Most recent studies have reported pacemaker dependency data from several time points, without any granularity to evaluate for 1) the earliest time point when patients displayed signs of freedom from pacemaker dependency and 2) if patients who were not pacemaker dependent at a particular time point had any changes in AV conduction or PPM requirement at subsequent follow up intervals. Furthermore, many studies followed patients for many years, and reported pacemaker dependency rates only at these late time points. These studies may fail to distinguish the effect of cardiac surgery on the conduction system from the natural history of AV conduction with aging. Studies assessing the status of AV conduction and pacemaker dependency at more proximal follow-up intervals would be more useful in determining whether ongoing permanent pacing is necessary. It follows that the optimal timing for PPM implantation in the postoperative setting has not been well established. Some authors have advocated for early PPM implantation in order to reduce the intense resource utilization and risk of complications associated with temporary pacing, which generally requires monitoring in the intensive care unit[11, 12]. Others have suggested a more conservative approach, allowing up to 6 days of temporary pacing prior to PPM placement[13]. Advocates for this conservative approach argue that unnecessary PPM implantation exposes patients to a small but significant risk of complications, in addition to the substantial financial burden of device implant and mandatory longitudinal follow-up. Existing guidelines reflect the paucity of evidence and lack of consensus on this topic. The 2008 ACC/AHA/HRS guidelines provide a class I indication for PPM implantation in “postoperative atrioventricular (AV) block that is not expected to resolve”, however there are no recommendations regarding identification of the patients at higher risk for delayed or non-resolution of postoperative block[14]. We believe that the future studies need to assess pacemaker dependency starting at an early time point (i.e. 30 days post-operative), and then follow patients longitudinally to better capture the time course of conduction system recovery, when it occurs.

The significant heterogeneity between studies with respect to the definition of the primary endpoint as well as the duration of follow likely contributes significantly to the variations in outcomes between studies. It also limits quantitative synthesis of the data and the ability to draw generalizable conclusions. Definitions of pacemaker dependency that require complete absence of artificial pacing over a 6 month interval are likely to be too strict, excluding patients who may do very well without artificial pacing. On the other hand, definitions requiring only 10 seconds of observation for intrinsic rhythm, with pacemaker set at 50 bpm in VVI mode may fail to identify patients who intermittently require ventricular pacing for prolonged periods. A consensus definition of pacemaker dependency would facilitate the generation of a more cohesive literature and improve our ability to compare outcomes between observational studies.

In an effort to identify specific populations of patients who might benefit most from permanent pacemaker implantation, several of the included studies have attempted to identify individual pre-operative, perioperative and postoperative variables that are independently associated with failure to recover AV conduction and persistent pacemaker dependency. The risk factor profile reported in the current literature varies considerably across different studies due to differing study designs and patient characteristics. Moreover, the variability in the definition of pacemaker dependency also limits comparison of risk factors across different studies. Well-conducted studies with uniform endpoints are needed in order to identify patients who truly require long-term pacemaker use and those that have a high likelihood of recovering conduction within a few months.

**Fig 2:**
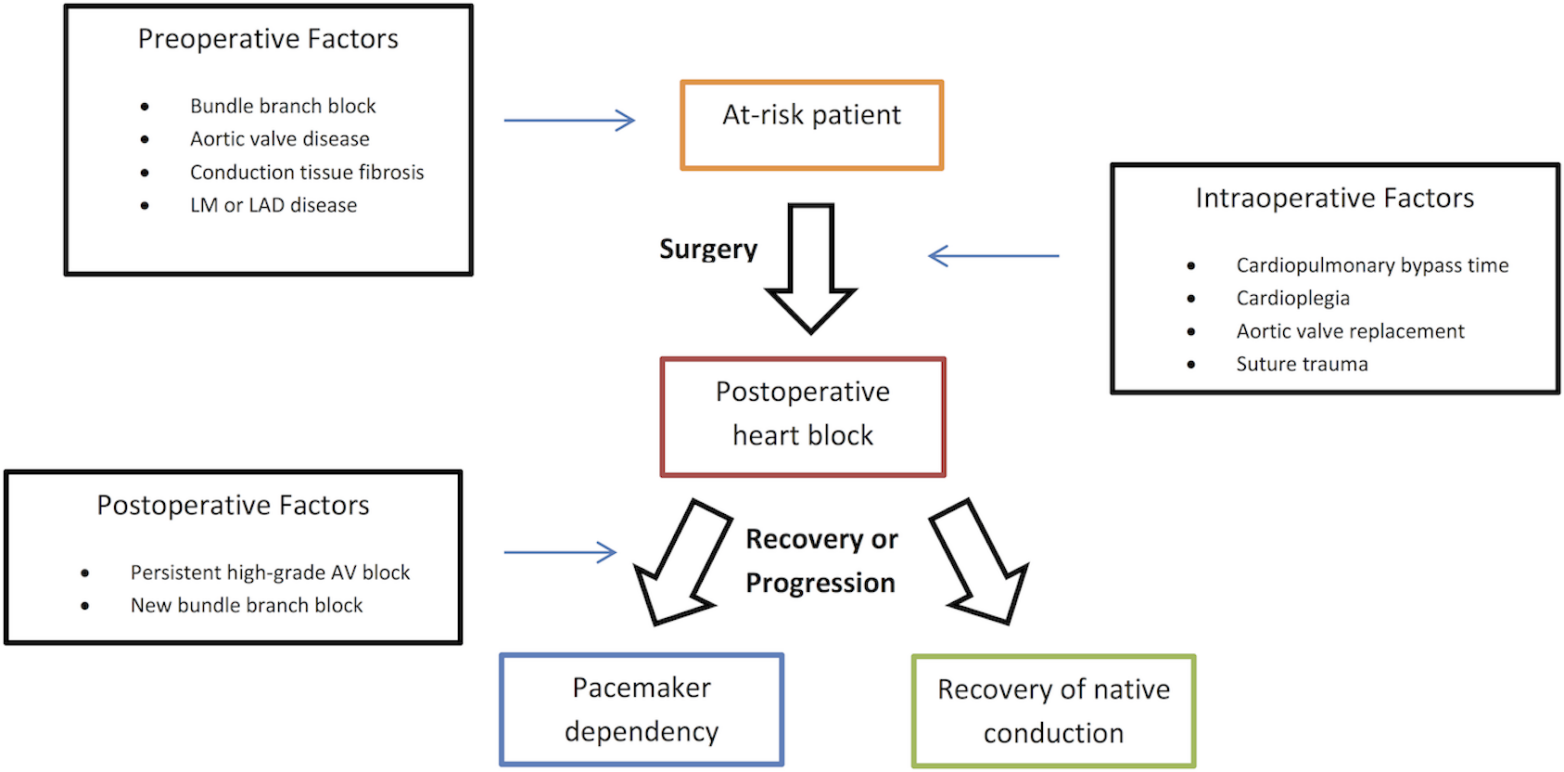
Pathophysiologic mechanisms underlying cardiac conduction system disease and recovery.

The synthesis of current studies along with previously published literature on post-operative conduction disease provides important clinical information to critically evaluate the proposed pathophysiological mechanism underlying the process of injury and recovery of the cardiac conduction system. Various mechanisms of damage to the conduction system have been proposed, and are likely to vary with the type of surgery. Further, the likelihood of recovery of native conduction is likely to be highly dependent upon the nature of the initial insult. Fig 2 summarizes the pathophysiological factors related to conduction system disease after cardiac surgery. Preoperatively, the presence of aortic valve disease, especially calcific aortic valve stenosis, is often a associated with underlying conduction system disease, even if not apparent on surface EKG[15]. Several studies have reported a higher incidence of heart block after surgical procedures for the treatment of aortic valve disease, including aortic valve replacement[16, 17]. The proximity of the aortic valvular apparatus to the His bundle provides a feasible anatomic substrate for this phenomenon. In a histologic study of patients who died within 30 days after surgical aortic valve replacement, Fukuda et al[18] identified three major morphologic lesions of the conduction system: (i) old lesions consistent with chronic degeneration and fibrosis; (ii) recent (perioperative) non-traumatic lesions, typically hemorrhagic; and (iii) recent (perioperative) traumatic lesions. Among traumatic lesions, laceration of conduction system fibers by sutures used to anchor the valve prosthesis were the most common etiology. The authors also described pressure from residual calcific material and impingement of the prosthetic valve seat upon conduction tissue as additional common traumatic lesions. This is further supported by the high rates of conduction disease in even sutureless valvular surgery. In a recent study, in patients undergoing sutureless aortic valve replacement, 9% developed completed AV block and17% required post-procedural PPM implantation[19]. It follows from these histologic data that patients who develop conduction disturbances after operations including aortic valve replacement might be less likely to recover native conduction, in view of the observation that traumatic injury to the conduction system is a common etiology in this population and is likely to cause permanent damage. Others have suggested that injury to adjacent tissues and resultant edema affecting conduction tissues studies might be responsible for transient conduction disturbances. In the studies reviewed herein, aortic valve replacement was frequently found to be associated with a higher incidence of postoperative pacemaker requirement, but an association with long-term dependency was only demonstrated in the study by Onalan et al[20].

It has also been postulated that perioperative ischemic injury to the conduction tissue, facilitated in part by left main or proximal left anterior descending (LAD) artery disease, plays a critical role in the genesis of postoperative heart block[21]. Studies attempting to link the presence of preoperative obstructive left main or LAD disease to development of heart block have yielded inconsistent results, however. None of the studies included in this review have demonstrated an independent association between coronary artery disease and late pacemaker dependency.

Periprocedural factors may play an important role in post-operative conduction disease and its reversibility. Historically, the contribution of cold potassium cardioplegic solution to postoperative heart block has been proposed[22]. This is related to the observation that the high concentration of potassium ions in cardioplegic solution acutely raises extracellular potassium concentration in the cardiac conduction tissue, thereby reducing the automaticity of the AV nodal cells and suppressing the excitability and conductivity of conduction system tissue. Multiple authors have suggested that the duration of time on cardiopulmonary bypass, an indirect measure of exposure to cardioplegic solution, might be associated with postoperative conduction disturbances. Several studies included in this review indeed suggest that prolonged cardiopulmonary bypass time (CPBT) is associated with long-term pacemaker dependency.

Individualizing pacemaker therapy based on a clearer understanding of patient and procedure-specific variables will allow for more appropriate use of permanent pacemakers, and may open avenues for devices requiring only temporary placement—so called ‘temporary-permanent’ pacemakers—for patients who are expected to recover native conduction in the short-term[23]. For patients expected to recover conduction, albeit at a later time-point, ongoing scrutiny over the clinical utility of the implanted device is prudent after the decision to implant a permanent pacemaker has been made. Current guidelines do not provide recommendations regarding removal of redundant pacemaker devices, or suggest restricting replacement of these devices when they are no longer clinically necessary. Long-term pacemaker use entails significant risk for the patient- both medically, with respect to iatrogenic device complications and financially, with the costs associated with continued need for clinical follow-up. Such a personalized approach might help reduce the risk of serious complications associated with permanent pacemaker implantation, and provide a more cost-effective solution for patients with only transient conduction disease.

In conclusion, the current evidence regarding post-cardiac surgery pacemaker use and long-term dependency is limited due to lack of a standardized study design. Future well-designed investigations utilizing uniform definitions and clinically useful endpoints will be instrumental in the exploration of this common clinical scenario in order to guide informed clinical decision-making.

## Supporting Information

S1 PRISMA Checklist PRISMA checklist for reporting systematic reviews and meta-analyses. (DOC)

S1 Fig List of search terms used for primary literature search. Search strings used for each database are listed individually.
(DOCX)

S1 Table Completed GRACE checklist for assessment of quality of observational studies. (DOCX)
