# Supplementary material for: Pacemaker Dependency after Cardiac Surgery: A Systematic Review of Current Evidence
Source: PLoS One. 2015 Oct 15;10(10):e0140340. doi: 10.1371/journal.pone.0140340 (PMC4607414; doi:10.1371/journal.pone.0140340)
Supplement: S1 Fig — Search strings used for each database are listed individually. (DOCX) [file pone.0140340.s002.docx]

**S1 Fig. List of search terms used for primary literature search.** Search strings used for each database are listed individually.

**(I)Medline:**

"Pacemaker, Artificial"[Mesh] OR "Cardiac Pacing, Artificial"[Mesh] OR "Heart Block"[Mesh] OR "Bradycardia"[Mesh] OR pacemaker*[Text Word] OR PPM[Text Word] OR CHB[Text Word] OR "complete heart block"[Text Word] OR "AV block"[Text Word]

AND

"Cardiac Valve Annuloplasty"[Mesh] OR "Heart Valve Prosthesis Implantation"[Mesh] OR "Heart Valve Prosthesis"[Mesh] OR "Coronary Artery Bypass"[Mesh] OR "Transmyocardial Laser Revascularization"[Mesh] OR "Pericardial Window Techniques"[Mesh] OR "Pericardiectomy"[Mesh] OR "Heart Valves/surgery"[Mesh] OR "Heart Valve Diseases/surgery"[Mesh] OR "Coronary Artery Disease/surgery"[Mesh] OR cardiac surgery[Text Word] OR "aortic valve replacement"[Text Word] OR AVR[Text Word] OR "mitral valve replacement"[Text Word] OR MVR[Text Word] OR CABG[Text Word] OR "heart surgery"[Text Word] OR "cardiothoracic surgery"[Text Word]

AND

"Observational Study"[Publication Type] OR "Epidemiologic Studies"[Mesh] OR follow up[Text Word] OR cohort[Text Word] OR case control*[Text Word] OR case series[Text Word] OR nonrandom*[Text Word] OR non random*[Text Word] OR prospective*[Text Word] OR retrospective*[Text Word] OR compare*[Text Word] OR compara*[Text Word] OR longitudinal[Text Word] OR "Time Factors"[Mesh] OR "Prognosis"[Mesh] OR "Risk Assessment"[Mesh] OR "Treatment Outcome"[Mesh] OR “Recovery of Function” [Mesh] OR dependence[Text Word] OR dependency[Text Word] OR dependent[Text Word] OR "risk factor*"[Text Word] OR "Risk Factors"[Mesh] OR predict*[Text Word] OR long term [Text Word] OR long-term [Text Word]

**(II) Web of Science**

TOPIC: (pacemaker*) OR TOPIC: ("cardiac pacing") OR TOPIC: ("heart block") OR TOPIC: (PPM) OR TOPIC: (CHB) OR TOPIC: ("complete heart block") OR TOPIC: ("AV block") OR TOPIC: (bradycardia)= 124,211

AND

TOPIC: ("cardiac valve annuloplasty") OR TOPIC: ("heart valve prosthesis") OR TOPIC: ("coronary artery bypass") OR TOPIC: ("transmyocardial laser revascularization") OR TOPIC: ("pericardial window technique*") OR TOPIC: (pericardiectomy) OR TOPIC: (heart valve surgery) OR TOPIC: (cardiac surgery) OR TOPIC: (cardiothoracic surgery) OR TOPIC: (AVR) OR TOPIC: ("aortic valve replacement") OR TOPIC: ("mitral valve replacement") OR TOPIC: (MVR) OR TOPIC: (CABG) OR TOPIC: (heart surgery) = 118, 201

AND

TOPIC: (observational) OR TOPIC: (epidemiologic) OR TOPIC: ("follow up") OR TOPIC: ("follow-up") OR TOPIC: (cohort) OR TOPIC: ("case control*") OR TOPIC: ("case series") OR TOPIC: (nonrandom*) OR TOPIC: ("non random*") OR TOPIC: (prospective*) OR TOPIC: (retrospective*) OR TOPIC: (compare*) OR TOPIC: (compara*) OR TOPIC: (longitudinal) OR TOPIC: ("time factor*") OR TOPIC: (prognosis) OR TOPIC: ("risk assessment") OR TOPIC: ("treatment outcome*") OR TOPIC: ("recovery of function") OR TOPIC: (dependence) OR TOPIC: (dependen*) OR TOPIC: ("long term") OR TOPIC: ("risk factor*") OR TOPIC: (predict*) OR TOPIC: ("long-term")= 8,530,396

Total=1713

**(III) EMBASE**

(('annuloplasty'/exp OR 'heart valve replacement'/exp OR 'heart valve prosthesis'/exp OR 'coronary artery bypass graft'/exp OR 'laser transmyocardial revascularization system'/exp OR 'heart surgery'/exp OR ((heart:ti OR heart:ab OR cardiac:ti OR cardiac:ab OR valve:ti OR valve:ab OR coronary:ti OR coronary:ab) AND (surgery:ti OR surgery:ab))) AND ('observational study'/exp OR 'epidemiology'/exp OR 'cohort analysis'/exp OR 'follow up'/exp OR 'case study'/exp OR 'case control study'/exp OR 'convalescence'/exp OR 'longitudinal study'/exp OR 'treatment outcome'/exp OR 'retrospective study'/exp OR (dependence:ti OR dependence:ab OR dependency:ti OR dependency:ab OR dependent:ti OR dependent:ab OR long AND term:ti OR long AND term:ab OR 'long term':ti OR 'long term':ab OR predictor:ti OR predictor:ab OR predictive:ti OR predictive:ab OR predict:ti OR predict:ab OR predicts:ti OR predicts:ab) OR 'risk factor'/exp OR 'prognosis'/de) AND ('artificial heart pacemaker'/exp OR (pacemaker:ti OR pacemaker:ab)) AND [english]/lim)

NOT (('annuloplasty'/exp OR 'heart valve replacement'/exp OR 'heart valve prosthesis'/exp OR 'coronary artery bypass graft'/exp OR 'laser transmyocardial revascularization system'/exp OR 'heart surgery'/exp OR ((heart:ti OR heart:ab OR cardiac:ti OR cardiac:ab OR valve:ti OR valve:ab OR coronary:ti OR coronary:ab) AND (surgery:ti OR surgery:ab))) AND ('observational study'/exp OR 'epidemiology'/exp OR 'cohort analysis'/exp OR 'follow up'/exp OR 'case study'/exp OR 'case control study'/exp OR 'convalescence'/exp OR 'longitudinal study'/exp OR 'treatment outcome'/exp OR 'retrospective study'/exp OR (dependence:ti OR dependence:ab OR dependency:ti OR dependency:ab OR dependent:ti OR dependent:ab OR long AND term:ti OR long AND term:ab OR 'long term':ti OR 'long term':ab OR predictor:ti OR predictor:ab OR predictive:ti OR predictive:ab OR predict:ti OR predict:ab OR predicts:ti OR predicts:ab) OR 'risk factor'/exp OR 'prognosis'/de) AND ('artificial heart pacemaker'/exp OR (pacemaker:ti OR pacemaker:ab)) AND [english]/lim AND [medline]/lim)

1765 7/11/14

((('artificial heart pacemaker'/exp OR 'heart pacing'/exp OR 'heart block'/exp OR 'bradycardia'/de OR 'bradycardia'/exp OR ('complete heart block':ti OR 'complete heart block':ab) OR ('av block':ti OR 'av block':ab) OR (pacemaker:ti OR pacemaker:ab)) AND ('annuloplasty'/exp OR 'heart valve replacement'/exp OR 'heart valve prosthesis'/exp OR 'coronary artery bypass graft'/exp OR 'laser transmyocardial revascularization system'/exp OR 'heart surgery'/exp OR ((heart:ti OR heart:ab OR cardiac:ti OR cardiac:ab OR valve:ti OR valve:ab OR coronary:ti OR coronary:ab) AND (surgery:ti OR surgery:ab)))) AND ('observational study'/exp OR 'epidemiology'/exp OR 'cohort analysis'/exp OR 'follow up'/exp OR 'case study'/exp OR 'case control study'/exp OR 'convalescence'/exp OR 'longitudinal study'/exp OR 'treatment outcome'/exp OR 'retrospective study'/exp OR (dependence:ti OR dependence:ab OR dependency:ti OR dependency:ab OR dependent:ti OR dependent:ab OR long AND term:ti OR long AND term:ab OR 'long term':ti OR 'long term':ab OR predictor:ti OR predictor:ab OR predictive:ti OR predictive:ab OR predict:ti OR predict:ab OR predicts:ti OR predicts:ab) OR 'risk factor'/exp OR 'prognosis'/de)) AND [english]/lim

NOT ((('artificial heart pacemaker'/exp OR 'heart pacing'/exp OR 'heart block'/exp OR 'bradycardia'/de OR 'bradycardia'/exp OR ('complete heart block':ti OR 'complete heart block':ab) OR ('av block':ti OR 'av block':ab) OR (pacemaker:ti OR pacemaker:ab)) AND ('annuloplasty'/exp OR 'heart valve replacement'/exp OR 'heart valve prosthesis'/exp OR 'coronary artery bypass graft'/exp OR 'laser transmyocardial revascularization system'/exp OR 'heart surgery'/exp OR ((heart:ti OR heart:ab OR cardiac:ti OR cardiac:ab OR valve:ti OR valve:ab OR coronary:ti OR coronary:ab) AND (surgery:ti OR surgery:ab)))) AND ('observational study'/exp OR 'epidemiology'/exp OR 'cohort analysis'/exp OR 'follow up'/exp OR 'case study'/exp OR 'case control study'/exp OR 'convalescence'/exp OR 'longitudinal study'/exp OR 'treatment outcome'/exp OR 'retrospective study'/exp OR (dependence:ti OR dependence:ab OR dependency:ti OR dependency:ab OR dependent:ti OR dependent:ab OR long AND term:ti OR long AND term:ab OR 'long term':ti OR 'long term':ab OR predictor:ti OR predictor:ab OR predictive:ti OR predictive:ab OR predict:ti OR predict:ab OR predicts:ti OR predicts:ab) OR 'risk factor'/exp OR 'prognosis'/de) AND [medline]/lim)

2996 7/11/14
